# Case report: osteogenesis imperfecta, internal mammary artery graft & nitinol clips

**DOI:** 10.1186/s13019-017-0685-2

**Published:** 2017-12-19

**Authors:** Ludovic Melly, Anne-Sophie Dincq, Claude Hanet, Benoît Rondelet

**Affiliations:** 1 Department of Cardiac, Vascular and Thoracic Surgery, CHU UCL Namur, Av. G. Thérasse 1, 5530 Yvoir, Belgium; 2 Department of Anesthesiology, CHU UCL Namur, Av. G. Thérasse 1, 5530 Yvoir, Belgium; 3 Department of Cardiology, CHU UCL Namur, Av. G. Thérasse 1, 5530 Yvoir, Belgium

**Keywords:** Osteogenesis imperfecta, Internal mammary artery, Nitinol clips

## Abstract

**Background:**

Osteogenesis imperfecta is a genetic disorder of connective tissue causing mostly left-sided heart valves and aortic root pathologies, but a coronary artery involvement reflecting an increased sensitivity to cardiovascular risk factors is also suspected in this patient population.

**Case presentation:**

We report a 38-year-old patient with an osteogenesis imperfecta and a typical presentation of an acute myocardial infarction. The coronary angiogram showed a coronary 3-vessel disease. The patient underwent a bypass grafting surgery with the internal mammary artery. The sternum was closed using four nitinol clips and had totally stabilized at 4 months with excellent bone healing.

**Conclusions:**

With the successful clinical outcome in this patient severely affected by its osteogensis imperfecta, we underline the safe use of the LIMA, if precaution is taken towards the sternal bone, and its closure with nitinol clips.

## Background

Osteogenesis imperfecta (OI) often called brittle bone disease, was first reported by Dr. Ekman in his medical thesis in Upsala at the end of the eighteenth century [1]. This orphan disease is a rare autosomal inherited genetic disorder of connective tissue with an estimated incidence of 1/20′000 births. Its defective synthesis of type 1 collagen is due several mutations in genes encoding the α1−/α2-chains divided into nine subtypes [2]; whereas the subtype II is lethal already in the perinatal period, the other forms can be found in older patients.

## Case presentation

**Fig. 1:**
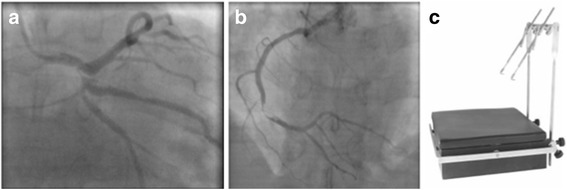
Coronary angiogram (**a**-**b**). Oschsner-Favalaro retractor (**c**)

A 38-year-old male, smoker, with a documented history of OI was referred with an evolving non-ST-elevation acute myocardial infarction (peak troponin value: 6.69 ng/mL; norm < 0.12). In addition the admission electrocardiogram showed a normal sinus rhythm without Q-waves nor typical repolarization disorders. Clinical examination revealed a short stature, the presence of blue sclera and a non-treated arterial hypertension. No bone abnormality was observed despite a childhood history of multiple traumatic fractures and a positive family history of OI with a son being similarly diagnosed. Echocardiography showed infero-posterior hypokinesia with preserved global left ventricular function. The coronary angiogram confirmed a severe 3-vessel disease (Fig. 1a-b).

**Fig. 2:**
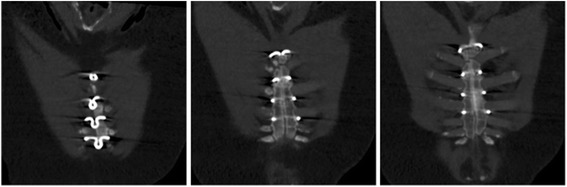
CT scan at 4 months

The patient underwent a triple coronary artery bypass grafting (CABG) on-pump with the left internal mammary artery (LIMA) on the left anterior descending coronary (LAD) as well as two separate vein grafts on the posterolateral branch and the distal right RCA. The LIMA was harvested with care using an Oschsner-Favalaro sternal retractor (Pilling^®^, Teleflex, Morrisville, USA), which lifts only one edge without compressing the opposite side (Fig. 1c). For sternal closure 4 nitinol clips (Flexigrips^®^, Praesidia, Bologna, Italy) were used. CT scan control at 4 months confirmed an excellent bone healing (Fig. 2).

## Discussion

Since type I collagen is an important constituent of the heart valves, the fibrous rings and the aortic wall, it is understandable that a defective synthesis is associated with left-sided valvulopathies. Nevertheless type I collagen is also present in other arteries including epicardial coronary arteries, and putatively contributes to their mechanical resistance to the phasic variations in pressure and blood flow. A Danish register-based cohort [3] has reported an increased risk of cardiovascular diseases and a statistically significant higher incidence of arterial hypertension compared to a matched population.

Independently of its possible role in the amplification of the sensitivity to risk factors of coronary atherosclerosis, OI influences the choice of revascularization technique. A reduced amount of type I collagen in coronary arteries is expected to decrease their resistance to stretch and to favor aneurysms formation or dissections, either spontaneously of in response to an iatrogenic aggression such as angiograms or percutaneous interventions. In-stent restenosis has previously been reported as early as 2 months even after drug-eluting stenting [4]. Conversely, tissue fragility and anticipated delayed sternal healing may considerably affect the risk of a surgical revascularization. In the present case, considering the 3-vessel disease involving the distal left main stem, the heart team unanimously agreed on the surgical revascularization.

In the literature, only a hand full CABGs were reported, all, to our knowledge, performed with vein grafts [5, 6]. In our institution, such a young patient would normally have been treated with bilateral mammary arteries as T-grafts. As a compromise we then decided to use only the LIMA on the LAD for his prognosis and vein grafts on the other vessels, in order to avoid complete de-vascularization of the sternal bone and potential healing problems. The sternal edges were treated with great care during sternotomy, LIMA harvesting and thereafter. Indeed the bone was osteoporotic and very friable but surprisingly the aorta displayed a common morphology and aortic cannulation could be performed as usual without any dissection or tearing complications. In regards to hemorrhage mostly caused by faulty platelet-endothelium interactions and clotting factor deficiency [7], we did not experience any anastomotic insufficiency neither distally on the coronary arteries neither central on the aorta. Nevertheless because of those concerns the second vein graft was anastomosed into the first one in order to minimize aortic touch and injury. No transfusion of red blood cells was required in our case and although not given, fresh frozen plasma and platelets had been made available perioperatively. For the closure 4 nitinol clips were used in order to keep a maximal compression without weakening the bone with supplementary holes as with trans-sternal standard wires. This device, very convenient for the closure especially of hemisternotomy [8], has not been used in this subgroup of patients previously. Both clinical and computer tomographic examinations confirmed the stability and the bone healing at 4 months and reinforce the pertinence of our choice.

## Conclusion

From the experiences summarized above, including the successful outcome, we underline the safe use of the LIMA, if precaution is taken towards the sternal bone, and its closure with nitinol clips.

## Abbreviations

CABG: Coronary artery bypass grafting
LAD: Left anterior descending coronary artery
LIMA: Left internal mammary artery
OI: Osteogenesis imperfecta
